# Defining the clinical pathway in cochrane diagnostic test accuracy reviews

**DOI:** 10.1186/s12874-016-0252-x

**Published:** 2016-11-10

**Authors:** G. Gopalakrishna, Miranda W. Langendam, Rob J. P. M. Scholten, Patrick M. M. Bossuyt, Mariska M. G. Leeflang

**Affiliations:** 1 Department of Clinical Epidemiology, Biostatistics and Bioinformatics, Academic Medical Center, University of Amsterdam, P.O Box 22660, 1100 Amsterdam, DD The Netherlands; 2 Cochrane Netherlands, Julius Center - UMC Utrecht Huispostnummer Str. 6.131 Postbus 85500, 3508 Utrecht, GA The Netherlands

**Keywords:** Cochrane systematic reviews, Diagnostic tests, Medical tests, Clinical pathways, Guidelines, Test accuracy studies

## Abstract

**Background:**

The value of a medical test depends on the context in which it might be used. Ideally, questions, results and conclusions of a diagnostic test accuracy (DTA) systematic review should be presented in light of this context. There is increasing acceptance of the value for knowing the impact a test can have on downstream consequences such as costs, implications for further testing and treatment options however there is currently no explicit guidance on how to address this. Authors of a Cochrane diagnostic review have recently been asked to include the clinical pathway in which a test maybe used. We aimed to evaluate how authors were developing their clinical pathways in the light of this.

**Methods:**

We searched the Cochrane Database of Systematic Reviews for all published DTA reviews. We included only those reviews that included a clinical pathway. We developed a checklist, based on the guidance in the Cochrane Handbook for DTA review authors. To this, we added a number of additional descriptors. We checked if the included pathways fulfilled these descriptors as defined by our checklist.

**Results:**

We found 47 reviews, of which 33 (73 %) contained aspects pertaining to a clinical pathway. The 33 reviews addressed the clinical pathway differently, both in content and format. Of these, 21 provided a textual description and 12 include visual and textual descriptions. There was considerable variation in how comprehensively review authors adhered to our checklist. Eighteen reviews (51 %) linked the index test results to downstream clinical management actions and patient consequences, but only eight went on to differentially report on the consequences for false negative results and nine on the consequences for false positive results.

**Conclusion:**

There is substantial variation in the clinical pathway descriptions in Cochrane systematic reviews of test accuracy. Most reviews do not link misclassifications (i.e. false negatives and false positive) to downstream patient consequences. Review authors could benefit from more explicit guidance on how to create such pathways, which in turn can help guide them in their evidence selection and appraisal of the evidence in the context of downstream consequences of testing.

**Electronic supplementary material:**

The online version of this article (doi:10.1186/s12874-016-0252-x) contains supplementary material, which is available to authorized users.

## Background

Medical tests are used in health care to provide information about the current or future status of a patient. Such tests include imaging tests, laboratory tests, physical examination and history taking or questionnaires. Tests are rarely used in isolation but typically form part of a test-treatment strategy; the results from testing guide clinical management. Ideally, recommendations about testing should be based on their ability to improve patient outcome [1, 2]. Yet direct evidence of the impact of a test or test strategies on patient outcomes is rare. For instance, the number of randomized trials that have evaluated test-treatment strategies is very small [2].

Many evaluations of medical tests focus primarily on the test’s accuracy: the ability of the test to correctly identify patients with the target condition. The results of such studies are typically reported as estimates of the test’s sensitivity and specificity. Estimates of test accuracy are rarely sufficient to judge the health benefits from testing. A test with 99 % test accuracy may not necessarily lead to improved patient outcomes if there is no effective treatment available, for example. It is also unclear to what extent a test with a specificity of 80 % will generate an improvement in patient outcome, or harm. The effects of accuracy therefore have to be put in context, by offering a description of the consequences of misclassifications and correct classifications, by linking testing to management actions and downstream patient outcomes via clinical pathways, also known as test-treatment pathways.

Mapping out clinical pathways describe the context in which testing may be used. It describes the setting and patients who would receive the test and can help us to understand whether the test of interest – the index test – is proposed as a triage test, an add-on test, or a replacement for an existing test or test strategy. Pathways help to define the possible downstream consequences of testing, as they also include the impact arising as a result of testing and/or the testing process. These may be outcomes that result from clinical management decisions following test results; the direct health effects of testing; the patients’ emotional, social, cognitive, and behavioural responses to testing; the legal and ethical effects of testing and/or the costs of testing [3]. In doing so, the pathway can help in assessing how the introduction of a new test may impact current diagnostic pathways.

Several organisations and initiatives that provide guidance on how to evaluate tests make reference to the development of such a pathway, with somewhat different approaches and levels of detail. Examples are the Evaluation of Genomic Applications of Practice and Prevention initiative (EGAPP), NICE (Diagnostic Assessment Programme), the US Preventative Task Force (USPSTF), the Agency for Healthcare Research and Quality (AHRQ) and the Cochrane Handbook for Systematic Reviews of Diagnostic Test Accuracy [3–5]. The terminology differs between the different organisations; some refer to a “care pathway”, others to “clinical pathway”, “clinical scenario” or “analytical framework” [4]. Explicit instructions for mapping such pathways appear to be limited or absent from these documents.

Evidence-based guidelines are often based on systematic reviews. Given that Cochrane systematic reviews are considered the most rigorously developed evidence syntheses, and because Cochrane Diagnostic Test Accuracy (DTA) review authors are required to develop a clinical pathway as part of their review since 2013, we systematically evaluated how authors were defining the clinical pathway in their reviews and which elements of the pathway they choose to describe. The Cochrane DTA handbook (ref) does provide a definition and a number of items the clinical pathway should include but no explicit guidance on how authors should go about working out such a pathway is provided. Through this evaluation, we hope to better understand the kind of guidance review authors would need in order to develop a complete and informative clinical pathway for a medical test or test strategy when synthesizing the available evidence.

## Methods

The definition and description of the clinical pathway in Cochrane DTA reviews was assessed using a checklist. The checklist was based on the criteria for developing a clinical pathway in the Cochrane DTA handbook [5]. In the handbook, the clinical pathway is defined as “how patients might present, the point in the pathway at which participants would be considered for testing with the index test (or tests), and the role of each index test. A diagram may be helpful, particularly if the pathway is complex. There are three further optional sub-headings to assist in this – prior test(s), role of index test(s) and alternative test(s)”.

To this, we added descriptors that can be used in creating a complete and informative clinical pathway description. These were based on the Patient-Index test-Comparator-Outcome (PICO) framework, other key references such as the AHRQ medical test evidence synthesis handbook [3] and the Grading of Recommendations Assessment, Development and Evaluation (GRADE) for diagnostic tests approach [3, 6], in addition to our combined professional experience in medical test evaluation. In total, the checklist consisted of three main categories: 1) the target condition, 2) the index test(s), and 3) the actual clinical pathway, combining aspects relating to the index test(s), existing test(s) and implications of testing on downstream consequences.

Two authors (GG and RS) piloted the draft checklist on two randomly selected Cochrane DTA reviews. When there was disagreement between the two assessors, these were discussed with two other co-authors (MWL and PMMB) to reach a resolution. After this process, we revised the criteria and definitions where needed.

The checklist was applied to completed and published Cochrane DTA reviews. The first Cochrane DTA review was published in 2008 [7]. Hence we searched the Cochrane Database of Systematic Reviews for all DTA reviews published from 2008 up to July 2015. In order to be included, a review either had to have an explicit clinical pathway heading, as required by the Cochrane DTA handbook [5], or report at least one of the following additional clinical pathway descriptors, as defined in our checklist (i.e. Additional file 1: Table S1, items 3.6-3.8). These descriptors relate to the linkage between the index test and downstream consequences, which we consider an essential feature in a clinical pathway description.

The requirement to include a clinical pathway description is part of the “Background” section of a DTA Cochrane review in the DTA Handbook, hence this section was first screened when making the selection of reviews to include. In addition, we also screened the “Introduction”, “Objectives” and “Methods” sections, as the piloting exercise revealed that some authors addressed relevant criteria in these sections as well.

We assigned two assessors (GG & PMMB, GG & MWL, GG &RS) for each DTA review. The assessors scored the review, in a double blinded fashion, with a yes or no for fulfilling each criterion on the checklist (Additional file 1). Disagreements between assessors were discussed. The aim of the duplicate assessment was to be objective as possible.

## Results

**Fig. 1:**
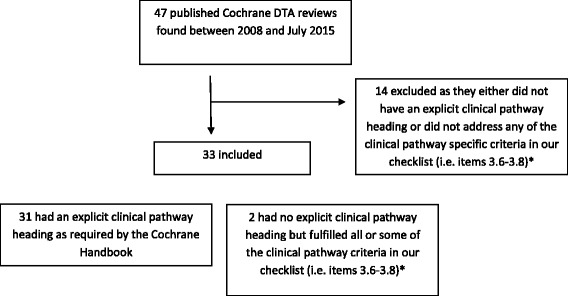
Search Results. *as defined in Additional file 1

**Fig. 2:**
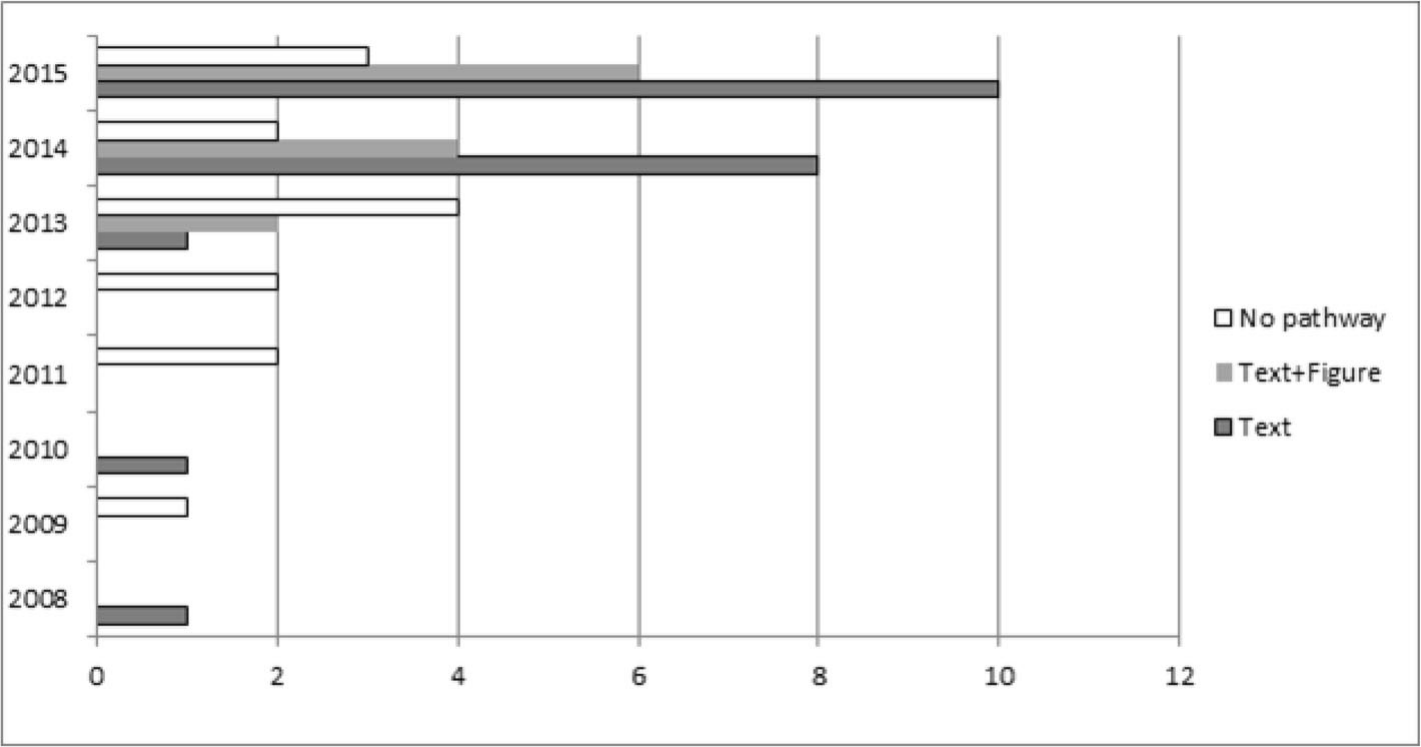
Distribution of the different ways in which the clinical pathway is described in Cochrane reviews published between 2008 and July 2015 (*n* = 47)

**Fig. 3:**
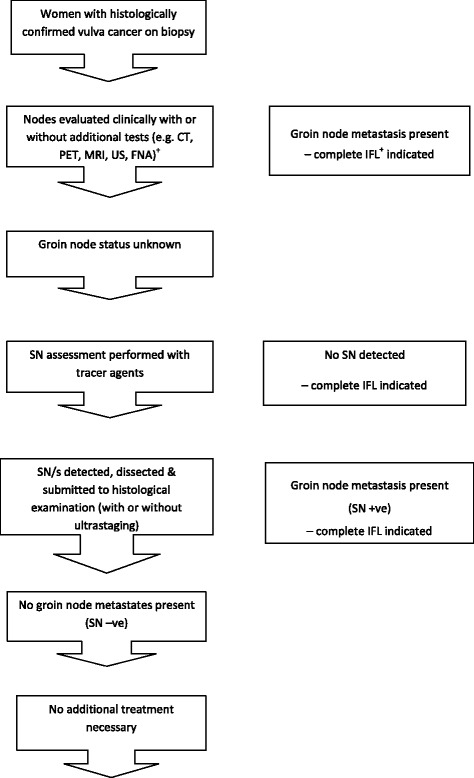
Clinical pathway of women with ≥ FIGO^+^ stage IB^+^ vulval cancer. +FIGO/IB International federation of Gynecology & Obstetrics classification; CT computed tomography; PET positron emission tomography; MRI magnetic resonance imaging; US ultrasound; FNA fine needle aspiration; IFL inguinofemoral lymphadenectomy. ++ Figure has been adapted from the following review entitled “Sentinel node (SN) assessment for diagnosis of groin lymph node involvement in vulval cancer (Review) The Cochrane Library 2014, Issue 6”

**Fig. 4:**
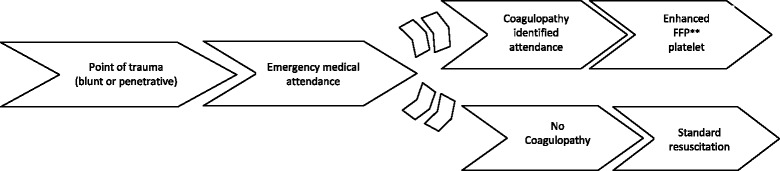
Clinical pathway for emergency department identification of trauma-induced coagulopathy. **FFP fresh frozen plasma. ***Figure has been adapted from the following review entitled “Thromboelastography (TEG) and rotational thromboelastometry (ROTEM) for trauma induced coagulopathy in adult trauma patients with bleeding (Review). The Cochrane Library 2015, Issue 2”

A total of 47 reviews from the Cochrane Database of systematic reviews published between 2008 and July 2015 (Fig. 1) were retrieved. Of these 47 reviews, 33 (70 %) either explicitly had a clinical pathway heading in their review or contained one or more of the essential clinical pathway descriptors (Fig. 1; Additional file 1, items 3.6-3.8). Additional file 1 presents the final checklist with definitions of the descriptors used to assess the pathways in these reviews. Figure 2 gives a breakdown of these 47 reviews. It also describes, for the 33 reviews with a pathway description, if the pathway was reported in the text and/or as a figure. The full list of reviews included in this study are available upon request. Figures 3 and 4 are examples of how authors may display clinical pathways in their Cochrane reviews. We have included these examples for the benefit of readers who may be unfamiliar with how such pathways may look like in a Cochrane DTA review when authors choose to display them as figures.

There is a visible increase in the number of Cochrane DTA reviews being published since 2008 (Fig. 2). Since the formal introduction of the “Clinical Pathway” in the Cochrane DTA Handbook as of January 2013 we also saw an increase in the number of reviews fulfilling this requirement. However, not all reviews from 2013 include a pathway. Out of the 14 reviews without a clinical pathway, 9 were published after 2013. Textual representation of the pathway (21/33; 64 %) seems to be the preferred way of describing the clinical pathway.

Table 1 provides the results of the extent to which the 33 reviews included in this study contained the descriptors. There was a wide distribution in terms of the number of reviews that contained both existing Cochrane descriptors and the additional descriptors we defined (indicated in italics) relating to the clinical pathway.

**Table 1:** Clinical pathway descriptors in Cochrane reviews (*n* = 33)

Clinical Pathway Descriptors	Ever reported in any section (%)
1 Target condition	
1.1 Is the target condition defined?	33 (100)
1.2 Are subcategories of the target condition defined?	21 (64)
1.3 Are the following aspects defined:	
frequency	29 (89)
severity	26 (80)
prognosis	24 (74)
possible treatment	29 (89)
*1.4 Is the relevance of the target condition explained in terms of downstream consequences?*	24 (74)
2 Index test	
*2.1 Is the purpose of the index test defined?*	33 (100)
2.2 Is the role of the index test defined?	19 (57)
2.3 Are test variations included?	15 (46)
*2.4 Are test specifications defined?*	16 (49)
3 Clinical pathway	
3.1 Is the existing pathway of patients defined as:	
how patients might present?	23 (69)
the point in the pathway where the index test might be considered	26 (80)
3.2 Are prior tests identified according to:	
clinical history and examination	27 (83)
healthcare setting	22 (66)
3.3 Is the patient or population receiving the index test identified according to:	
Clinical Pathway Descriptors	Ever reported in any section (%)
stage or disease severity	16 (49)
age	12 (37)
gender	12 (37)
has the patient received single or multiple assessments prior to index testing	21 (63)
3.4 Are alternative tests described	27 (83)
*3.5 Is it explicit how the index test/strategy compares to the existing test/strategy? Items to consider are:*	
* are existing tests described*	20 (60)
* is it clear if the existing test is current practice and/or reference standard*	*19 (57)*
*3.6 Is the impact of the index test on downstream clinical management action(s) explicit?*	17 (51)
*3.7 Is it explicit how index test/strategy when compared to current practice affects downstream outcomes?*	17 (51)
*3.8 Are the downstream consequences differentially described according to the 4 test accuracy categories (TP, TN,FP, FN) as follows:*	
* TP : is there effective treatment or further testing needed?*	*14 (43)*
* TN: re-testing, FU and/or intervals*	9 (26)
* FP: consequences to this group*	9 (26)
* FN: consequences to this group*	8 (23)

Normal font refer to existing Cochrane criteria in the Cochrane DTA Handbooks*; italics* refer to additional descriptors included by us

The main category “Target Condition” and its corresponding subcategories were defined in majority of the reviews (between 64- 100 %, depending on the subcategory; Table 1). The relevance of the target condition, explained in terms of its impact on downstream consequences, was reported in 24 reviews (74 %). For instance, in the review by Allen et al., the target condition was the inability to perform curative resection of pancreatic and periampullary cancer (’unresectable’ cancers). By determining which patients have unresectable cancers, these patients can be spared laparotomy and consequently, this can help decrease associated morbidity and costs due to unnecessary laparotomy (Additional file 1).

For the main category “Index Test”, the purpose of the index test (additional descriptor) was defined in all 33 reviews (100 %), but the role of the test (i.e. add-on, triage, replacement; Cochrane descriptor) was reported in only 19 (57 %) of the reviews. A little less than half of the reviews reported on test variations and specifications: 15 (46 %) and 16 (49 %) respectively.

For the third main category, the “Clinical Pathway”, reporting of descriptors ranged from 8 reviews to 27 reviews (percentage range: 23 % to 83 %; Table 1). Patient characteristics (disease stage, severity, age and gender) were relatively infrequently reported (37 %–49 %). For the items on the checklist relating to linking the index test to downstream consequences (items 3.5–3.7) more than 50 % (17–20 reviews) of review authors addressed these criteria in their review despite this not being an explicit Cochrane descriptor. However, linking the downstream consequences differentially to test accuracy categories (true positives, true negatives, false negatives, false positives; Item 3.8, Table 1) was infrequently reported (23 %–43 % or 8–14 reviews) (Figs. 3 and 4).

## Discussion

We observed an upward trend in the number of Cochrane DTA reviews fulfilling the “clinical pathway” requirement, as required by the Cochrane DTA handbook. However, not all reviews published since 2013, when the inclusion of a clinical pathway became a requirement in Cochrane DTA reviews, include a description of the pathway. Out of the 14 reviews without a clinical pathway, 9 reviews were published after 2013.

Amongst the 33 reviews that fulfilled the Cochrane pathway requirements, there is a wide variety in how authors provided a pathway description. The range of fulfilment of our criteria ran from as high as 100 % to as low as 23 %, depending on the descriptor, with the lowest percentage for the criteria on linking testing consequences to downstream outcomes. Textual description is the most frequent choice among authors when describing the clinical pathway. The two example pathways (Figs. 3 and 4) included in this study demonstrate the extent to which clinical pathways can differ in terms of comprehensiveness of descriptors defined in our study.

Our assessment of the development of the clinical pathway was restricted to the Background, Clinical Pathway and Methods sections, as proposed by the DTA handbook. This may be a limitation of our study as we did not look at whether review authors tried to address the consequences of testing on downstream outcomes when discussing the implications of the index test for research and practice. It is also unclear from this study if the authors of the nine DTA reviews from 2013 did not include a clinical pathway despite this already being a Cochrane requirement due to lack of clarity in guidance or due to other reasons. In this study, only Cochrane DTA reviews were included. Therefore our findings may not be applicable to other diagnostic test accuracy reviews.

The results of our evaluation however imply that amongst the review authors who did comply, some are clearly going beyond the Cochrane clinical pathway definition to include descriptors that are currently not in the Cochrane definition of a clinical pathway. For example, more than 50 % of review authors linked the consequences of testing to downstream consequences (17/33; 51 %). However, less than 50 % of reviews (23–46 %) went a step further, to differentially link the test accuracy categories to the corresponding downstream consequences of correct and incorrect classifications based on the test’s results.

While our findings are encouraging, and might be indicative of an awareness among review authors to go beyond test accuracy, more awareness and explicit guidance is probably needed when it comes to differentially addressing the impact an index test may have on downstream consequences as a result of being classified as a true positive, true negative, false positive or false negative.

Both USPSTF and AHRQ [3, 4] have highlighted the usefulness of developing the pathway in evidence synthesis for developing recommendations about testing, as a means of clarifying the specific questions a medical test review or guideline should focus on. This can help guide the eventual evidence selection and assist in assessing the impact a new test may have on clinical management decisions and other patient outcomes. In most reviews in our study however, integration of the pathway in the aim and purpose of the review was missing, which may indicate a lack of clear understanding among review authors of the usefulness of including such pathways in their reviews.

Explicit guidance with worked examples could be one way of helping future authors develop clinical pathways that address more comprehensively all relevant aspects in a more standardized and explicit manner. Depending on the role and purpose of the index test, and the type of existing test strategy, each pathway may be different. Providing users with both guidance and many worked examples would be one way to provide clarity on this complex topic. In-depth interviews of guideline developers involved in making recommendations about medical tests attest to the importance of including pathways in medical test guideline development but they struggle on how and when such pathways should be developed during the guideline process [8]. This emphasizes the clear need for better guidance on how such pathways can be created and used to address the downstream impact a test may have on clinical management decisions and patient consequences as a result of receiving the test

We anticipate developing such guidance is best addressed via an iterative process, involving further and extensive user testing to guide the development of the specific criteria needed to develop the clinical pathway in test accuracy reviews. It may also be helpful to conduct a qualitative analysis among review authors, to further identify challenges and issues they may have faced while developing the pathway. This can help Cochrane in providing more explicit guidance to its authors.

## Conclusion

The increasing presence of pathways in the manuals of organisations focussed on evidence synthesis and guideline development for medical tests such as AHRQ, GRADE for diagnostics and NICE [3, 6, 9–11], indicate there is a growing awareness on the importance of such pathways in medical test evidence evaluation. This fits with our conclusion that Cochrane review authors are also demonstrating an increased awareness on such pathways in their reviews. Yet there seems to be a need for clear explicit guidance on how such pathways can be created and linked in the development of such reviews. We think future work could focus on in-depth interviewing of review authors which can help us understand reasons behind observations made in this study and help in determining more precisely the problems authors face. This can aid in developing targeted solutions in order to better help authors tackle the inclusion of pathways in their reviews.

## Additional file

Additional file 1: The clinical pathway descriptors – full explanation with examples. (DOC 72 kb)

## Abbreviations

AHRQ: Agency for Healthcare Research and Quality
DTA: Diagnostic test accuracy
EGAPP: Evaluation of Genomic Applications of Practice and Prevention
GRADE: Grading of Recommendations Assessment, Development and Evaluation
NICE DAP: National Institute for Health and Care Excellence Diagnostic Assessment Programme
USPSTF: USe Preventative Task Force
