# Supplementary material for: Defining the clinical pathway in cochrane diagnostic test accuracy reviews
Source: BMC Med Res Methodol. 2016 Nov 10;16:153. doi: 10.1186/s12874-016-0252-x (PMC5103389; doi:10.1186/s12874-016-0252-x)
Supplement: Additional file 1: — The clinical pathway descriptors – full explanation with examples. (DOC 72 kb) [file 12874_2016_252_MOESM1_ESM.doc]

**Supplementary Table - The clinical pathway** descriptors – full explanation with examples

| **Clinical pathway descriptor** | **Definition** | **Example** |
| --- | --- | --- |
| **1 Target condition** |  |  |
| 1.1 Is the target condition defined? | This is defined as a particular disease or disease stage that the index test is intended to identify | In the review by Lenza et al, the target condition was defined as any rotator cuff tears (i.e. partial or full thickness) in people with shoulder pain for whom surgery is being considered |
| 1.2 Are subcategories of the target condition defined? | Sub categories maybe different malignancy stages as in cancer or it may be refining a group of patients with the target condition into different treatment groups (e.g. differentiating between breast lumps that are benign and those that are malignant) | Lenza et al defined subcategories as rotator tear cut offs of partial or full thickness; In the review by Fage et al, this was defined as Alzheimer’s disease dementia and all related dementias such as dementia with Lewy bodies, Parkinson’s disease dementia, dementia with mixed etiology or vascular dementias |
| 1.3 Are the following aspects defined: |  |  |
| frequency | May be defined as incidence, prevalence | Fage at al defined this as “Alzheimer’s disease and related forms of dementia are common  among older adults with a prevalence of 8% in individuals over the age of 65 years, increasing to a prevalence of approximately 43%in adults aged 85 years and older” |
| severity | May be defined as mild moderate or severe, or stages in cancer etc | Macey et al defined this in their review as oral cancer and potentially malignant disorders of the lip and oral cavity, with any level of dysplasia (mild, moderate or severe) |
| prognosis |  | Macey et al defined the prognosis of oral cancer diagnosed at a late stage as poor with the five-year survival following diagnosis at  around 50% but can be curable if detected early |
| possible treatment(s) |  | Macey et al introduce a number of treatments briefly, such as surgery and radiotherapy for oral cancer treatment |
| **Clinical pathway descriptor** | **Definition** | **Example** |
| *1.4 Is the relevance of the target condition explained in terms of downstream consequences?* | *Downstream consequences are defined as outcomes related to the test results or testing process. These may be 1) direct health effects*  *( i.e. target condition), 2)emotional, social, cognitive, behavioural responses as a result of testing, 3) legal or ethical effects of testing, 4) costs of testing* | *In the review by Allen et al,the target condition being diagnosed is unresectable pancreatic and periampullary cancers. By determining which patients have such cancers, they can be spared having to undergo laparotomy and consequently, this can help decrease the costs and morbidity associated with unnecessary laparotomy. In the review of early diagnosis of schizophrenia, early diagnosis can lead to early intervention which is important in avoiding or postponing damaging relapses and/or the need for prolonged use of medications which have side effects* |
| **2 Index test** |  |  |
| *2.1 Is the purpose of the index test defined?* | *Purpose of a test may be diagnostic, prognostic, monitoring etc* | *In the review by Macey et al, the index test was diagnostic or in secondary care setting could be prognostic* |
| 2.2 Is the role of the index test defined? | Role of a test is defined as triage, replacement, add-on in comparison to existing test(s) | The index test in the primary care setting of the review by Macey et al was an adjunct triage test to the existing test |
| 2.3 Are test variations included? | Variations may be defined as whether there are different manufacturers of a test, who will be operating and interpreting the test, and whether more than one threshold will be considered | In the review by Lawrie et al, the index tests are tracer agents used to detect sentinel nodes for histological assessment, hence there can be different techniques and types of tracer agents used for this purpose. In the review by Zhang, the 11C-PIB-PET scan is an index test for the detection of Abeta deposition in the brain region of interest and different threshold positivity were assessed depending on what was defined as test threshold in the primary studies |
| *2.4 Are test specifications defined?* | *Test specifications include items such as resource requirements, training, translations, specialised equipment or conditions etc* | *In the review by Soares, the index test, FRS, for early diagnosis of schizophrenia was identified as needing low technology and minimal training hence a suitable test for low resource settings* |
| **Clinical pathway descriptor** | **Definition** | **Example** |
| **3 Clinical pathway** |  |  |
| 3.1 Is the existing pathway of patients defined as: |  |  |
| how patients might present | This may be defined in different ways e.g. if patients present with clinical symptoms or as possible or probable cases | In the review by Macey et al, patients were defined as older than 16 years of age with clinically evident oral lesions identified by frontline clinicians |
| the point in the pathway where the index test might be considered | This is defined as the point in the pathway where the index test is considered for use i.e. in primary, secondary, tertiary, or community setting | In the same review, the point in the pathway is defined as during conventional oral examination at the dentist or by a frontline clinician |
| 3.2 Are prior tests identified according to: |  |  |
| clinical history and examination | Defined as whether the patient has undergone clinical examination and/or history taking which can be considered as prior testing | See above example |
| healthcare setting | Healthcare setting may be community, primary, or secondary care. This may be used as a surrogate measure of the type and number of tests patients might have received prior to receiving the index test | Macey et al, the setting is defined as primary care/ dental visit |
| 3.3 Is the patient or population receiving the index test identified according to: |  |  |
| stage or disease severity | May be defined as mild, moderate or severe disease, stages in cancer etc | In the same review by Macey et al, this is specified as patients having clinically evident lesions, Mocellin et al defined this as early or advanced stage gastric cancer |
| age |  |  |
| gender |  |  |
| has the patient received single or multiple assessments prior to index testing | This may be described or stated as the number of prior tests, if any, patients have received before index test | In the same review by Macey, patients are specified as not having had any other tests besides the conventional oral examination prior to index testing |
| 3.4 Are alternative tests described | Defined as other diagnostic tests and strategies that could be used in clinical practice, but that aren’t evaluated in the review | In the review by Mocellin, CET, MRI and PET are defined as alterative tests |
| *3.5 Is it explicit how the index test/strategy compares to the existing test/strategy? Items to consider are:* | *Existing test refers to current practice which may or may not be the reference test. Descriptions may include the no. of existing tests, role and purpose of existing test(s) and if it/they is/are the reference standard and/or current practice* |  |
| **Clinical pathway descriptor** | **Definition** | **Example** |
| *are existing tests described* |  | *Mocellin et al describe the sensitivity and specificity of CT which is the existing test used in gastric cancer diagnosis* |
| *is it clear if the existing test is current practice or reference standard* |  | *In the above review, it is clear that the existing test, CT, is not the reference standard* |
| *3.6 Is the impact of the index test on downstream clinical management action(s) explicit?* | *Clinical management actions refer to decisions around further testing, follow-up, treatment etc* | *In the review by van der windt et al, downstream clinical management decisions were identified as those around the clinical management plan relating to further referral or surgical intervention. Walsh et al identified these as decisions involving referral for further*  *investigation in the form of*  *examination/biopsy by a specialist in oral medicine or oral surgery at a secondary or tertiary clinic* |
| *3.7 Is it explicit how index test/strategy when compared to current practice affects downstream outcomes*?* | *This is a comparison in terms of the impact between the index test/strategy and what is currently being done on downstream consequences (Item 1.4 defined downstream consequences)* | *In the review by Rutten et al, the index test was identified as being less invasive and carrying less complications than the alternative existing test.* |
| *3.8 Are the downstream consequences* differentially described according to the 4 test accuracy categories (TP, TN,FP, FN)** as follows:* |  |  |
| *TP > is there effective treatment or further testing needed?* |  | *In the review by Schmidt et al, on the use of PET CT for detection of resectable non-small cell lung cancer, TP are those patients with no spread to the mediastinum who may therefore be candidates for resection, and patients with either distant or mediastinal metastases, or both, that may need to be biopsied before their treatment plan*  *can be developed.* |
| **Clinical pathway descriptor** | **Definition** | **Example** |
| *TN> re-testing, follow up and/or intervals* |  | *In the same review, TN (i.e., surgical staging). Those*  *patients who are found to have unresectable NSCLC will usually*  *have had their cancer stage pathologically confirmed by a number*  *of other tests that are considered suitable for the location of the*  *affected lymph node(s).* |
| *FP> consequences to this group* |  | *Schmidt et al in their review of the use of PET CT for detection of resectable non-small cell lung cancer, identified false negative outcomes of PETCT*  *to apply only to nodes that are not significantly enlarged*  *on (a prior) CT, as enlarged nodes should be biopsied. False positives*  *are of a lesser concern since they should always be followed by a further test to confirm.* |
| *FN> consequences to this group* |  |

Items in italics refer to additional descriptors included by the authors that are not in the Cochrane DTA handbook currently

*see item 1.4 for definition; **TP = true positives; TN= true negatives; FP=false positives; FN= false negatives

***References***

*Magnetic resonance imaging, magnetic resonance arthrography and ultrasonography for assessing rotator cuff tears in people with shoulder pain for whom surgery is being considered (Review). Lenza M et al. Cochrane Database Sys Reviews; 2013; 9*

*Mini-Cog for the diagnosis of Alzheimer’s disease dementia and other dementias within a community setting (Review). Fage BA et al. Cochrane Database Sys Reviews; 2015; 2*

*Diagnostic tests for oral cancer and potentially malignant disorders in patients presenting with clinically evident lesions (Review). Macey R et al. Cochrane Database Sys Reviews; 2015;5*

*Diagnostic accuracy of laparoscopy following computed tomography (CT) scanning for assessing the resectability with curative intent in pancreatic and periampullary cancer (Review). Allen VB et al. Cochrane Database Sys Reviews; 2013;11*

*Sentinel node assessment for diagnosis of groin lymph node involvement in vulval cancer (Review). Lawrie TA et al. Cochrane Database Sys Reviews; 2014;6*

*First rank symptoms for schizophrenia (Review). Soares-Weiser K et al. Cochrane Database Sys Reviews;2015;11*

*Diagnostic accuracy of endoscopic ultrasonography (EUS) for the preoperative locoregional staging of primary gastric cancer (Review). Mocellin S, et al. Cochrane Database Sys Reviews; 2015;2*

*Physical examination for lumbar radiculopathy due to disc herniation in patients with low-back pain (Review). van der Windt DAWM, et al. Cochrane Database Sys Reviews; 2010;2*

*Laparoscopy for diagnosing resectability of disease in patients with advanced ovarian cancer (Review). Rutten MJ, et al. Cochrane Database Sys Reviews; 2014;2*

*PET-CT for assessing mediastinal lymph node involvement in patients with suspected resectable non-small cell lung cancer (Review). Schmidt-Hansen M et al. 2014*
